# Care and Treatment of Children's Teeth

**Published:** 1886-08

**Authors:** Langdon S. Chilcott

**Affiliations:** Bangor, ME.


ARTICLE V.

### CARE AND TREATMENT OF CHILDREN'S TEETH.

BY LANGDON S. CHILCOTT, D. D. S., BANGOR, ME.

(Read before the Florida Dental Association.)

In response to your President's very polite invitation to contribute a paper to be read at your convention, I select this subject—not because I expect to present any new ideas, but as it is one of practical importance, and given very little thought by most parents and very many dentists, and one which will, at least, have a brief review.

In treating these young patients, it is first necessary to have the co-operation of the parents, or whoever may be most interested; for only by such help can we depend on the fulfilment of our instructions.

Surely, we can do nothing in our practice that will be of more permanent good than to establish principles of cleanliness with our young patients in regard to their teeth.

As soon as a child has teeth, it should be the daily duty of the mother to care for them, seeing that perfect cleanliness is maintained, and as soon as practicable, teach it a judicious use of the tooth brush, floss silk and tooth-pick, and provide it with a suitable dentifrice.

Thus, early becoming habitually attentive to the teeth, it will not feel comfortable unless they are cleaned after each meal.

Too much care cannot be taken to see that the teeth are properly used and not injured at meals, as mere ornaments, by bolting the food without properly masticating or making an emulsion of it, as is done in some cases, by drinking water or milk, or what is worse, tea or coffee with almost every mouthful. Evil effects, caused by soaking the food in this way, and preventing a proper mixture of saliva, are too well known to more than mention here. It seems to be a principle of nature not to maintain anything for which it has no use, and it certainly looks reasonable that if we disregard the use of the teeth, especially the temporary set, nature will not endow the permanent in their formation and development as it otherwise would.

It cannot be expected by hygienic treatment to build up a fine denture where there is a predisposition to decay, but I believe much can be done for the permanent set and for the teeth of succeeding generations, by *caring for* and *using* the temporary sets of the present.

Those people who delight in talking of their experience in the dental chair, and for the sake of representing themselves as martyrs, by giving in magnified detail all the pains they have endured, and describe the horrid instruments and "piles of appliances" which, *they say*, they have seen and felt in the office of their famous dentist, make an irreparable mistake when they indulge in conversation of this nature before children, for their little ears are wide open to everything that is said, and it cannot fail to create a fear that will, when they visit their dentist, be distressing to themselves and perplexing to the operator.

It would be a mistake to deceive them with the idea that they will enjoy the dental chair.

Let mothers see to it, then, that their children are not intimidated, so when they are taken to the dentist (which should be as early as the second year, and earlier if the teeth show signs of decay), they may go with as much unconcern as possible. As the temporary teeth are only intended to last a brief period, it is argued by many dentists that it is not worth while to give them any attention more than to extract an offensive one when it causes pain. Such teachings and practice are wrong, and cruel to the children, and, I am glad to say, are fast being buried in the cemetery of dental antiquities.

In asserting that sufficient care and treatment should be given the temporary teeth to assist them in maintaining their places their allotted time, I am aware I stand in direct opposition (to his patients, at least), of a certain well known dentist, yet this same man is *Professor of Operatire Dentistry* in one of the best dental colleges in this country.

I hope I am not too presumptuous in denouncing the practice of some of my senior brethren, but believe that if one is to err on this subject, it may not be by saying too much.

To ensure success with children, it is of the greatest importance to gain their confidence, and when this is accomplished the battle may be considered won. The only way to hold such hard earned ground is to deal squarely with them. That stale lie, "it won't hurt," and the forcep up the sleeve, are passai. Victims of this old-fashioned "funny business" are those who hate to have their teeth even looked at.

Handle the children, then, with all possible gentleness.

Better, by far, insert a very temporary filling that must be replaced in a few weeks, than to thoroughly excavate and fill a very sensitive cavity in one of these small teeth.

Oxyphosphate of zinc, gutta percha, amalgam and tin, are the materials best adapted to the filling of temporary teeth. The operator must be governed by conditions, which agent should be employed, bearing in mind that the relatively large pulp must be protected from thermal changes, and fillings for large crown cavities must be able to resist the force of mastication as well as a poor conductor of heat. As less pain is caused by excavating a cavity when dry, this condition should be maintained as much as possible, but as such operations are usually brief, and it requires so little time when everything is ready to fill the cavity, the napkin and bibulous paper, in most cases, is sufficient. I have never yet found it necessary to apply rubber dam to a deciduous tooth.

In cases of an exposed pulp, a pellet of cotton, saturated with creosote, and placed in the cavity, carefully sealed, allowing it to remain in a few days, is all that is required.

After the devitalization of the pulp has been accomplished, as much of it as possible removed, its cavity filled with crude cotton saturated with creosote, or carbolic acid, and the crown filled with any material that will insure cleanliness, no further trouble need be expected from that tooth during the time it should remain in the mouth.

When a deciduous tooth, with a dead and putrescent pulp is presented for treatment, the first step is to open the pulp chamber freely, and if, after allowing it to remain long enough for the soreness to entirely disappear, it promises to be comfortable, the pulp cavity may be disinfected and the tooth filled with any material that may be best adapted to the case; if not, it is best to remove all decay and enough of the crown to make it clean, then let it alone.

I would at once extract a filthy, abscessed root, or one that could not be easily put and kept in a comfortable condition.

But I would prefer all roots to remain in position their appointed time; not for fear of any change of shape in the jaw by their premature loss, but if the roots are not absorbed, by extracting them at the proper time, it is much easier for the new tooth to struggle to the surface.

The sixth year molar, although belonging to the permanent set, in its treatment before the twelfth year, comes in, properly, with children's teeth.

As soon as decay is seen, the cavity should be excavated and filled, so the tooth will be comfortable, and in *all cases* this tooth should be retained until the eruption of the second molar may be expected, when its destiny can best be determined by an intelligent dentist.—*Southern Dental Journal.*

